# The impact of vision impairment on depressive symptoms among older adults: The mediating role of dispositional optimism

**DOI:** 10.1371/journal.pone.0328053

**Published:** 2025-07-09

**Authors:** Xi Ji, Yujie Zhang

**Affiliations:** 1 School of International and Public Affairs, Shanghai Jiao Tong University, Shanghai, Xuhui District, China; 2 School of Public Policy and Administration, Chongqing University, Chongqing, Shapingba District, China; Saint Francis University, HONG KONG

## Abstract

**Background:**

Vision impairment, beyond constituting a sensory deficit, fundamentally disrupts older adults’ autonomy, emotional regulation, and social participation. These disruptions increase vulnerability to depressive symptoms. While the adverse mental health consequences of sensory decline are well-documented, the psychological mechanisms underlying this association remain insufficiently examined. Dispositional optimism—a stable cognitive-affective trait characterized by generalized positive future expectations—may serve as a key mediator, promoting resilience through adaptive appraisal and emotional regulation processes.

**Objective:**

This study examines the mediating role of dispositional optimism in the relationship between vision impairment and depressive symptoms among older adults, and explores how its buffering effects vary by perceived control and socioeconomic resources. The findings aim to inform the integration of psychological resilience frameworks into geriatric mental health care and vision rehabilitation programs.

**Methods:**

This study used panel data from 7,205 older adults in the 2014 and 2018 waves of the Chinese Longitudinal Healthy Longevity Survey (CLHLS). Depressive symptoms were assessed using a harmonized CES-D scale, vision impairment through a field-based functional vision screening, and dispositional optimism via a 5-point Likert item. Ordered logit regression and bootstrapped mediation models were employed to estimate both direct and indirect effects, with subgroup analyses conducted to test for moderation by perceived control and socioeconomic status.

**Results:**

Vision impairment was significantly associated with increased depressive symptoms (β = −0.218, p < 0.001). Dispositional optimism mediated 24.7% of this effect (indirect effect = −0.004, p < 0.01), indicating its role as a psychological buffer. The protective function of optimism was more pronounced among individuals with higher perceived control (β = −0.135, p < 0.05), higher educational attainment (β = 0.264, p < 0.001), and better economic status (β = 0.210, p < 0.001). Notably, even among individuals with low perceived control, optimism continued to significantly mitigate depressive symptoms (indirect effect = −0.002, p < 0.05).

**Conclusion:**

These findings reveal the importance of incorporating psychological resources into vision care strategies for older adults—particularly the oldest-old and those in socioeconomically disadvantaged contexts. Interventions aimed at fostering dispositional optimism, such as cognitive-behavioral techniques, may enhance emotional resilience, reduce depression risk, and improve quality of life in aging populations experiencing sensory decline.

## Introduction

Vision impairment, encompassing a spectrum from mild refractive errors to irreversible blindness, is not merely a physiological deficit but a multidimensional challenge with profound social and psychological consequences [1,2]. Age-related visual disorders—such as cataracts, macular degeneration, and diabetic retinopathy—are becoming increasingly prevalent in rapidly aging societies such as China, positioning vision impairment as an urgent global public health concern [3,4].

Between 1990 and 2021, the number of individuals affected by age-related macular degeneration worldwide more than doubled, rising from 3.6 million to over 8 million, with projections suggesting a further increase to 13.9 million by 2050 [5]. Among older adults—particularly the oldest-old and those in socioeconomically disadvantaged regions—vision impairment intensifies structural health inequalities by restricting physical mobility, curtailing social participation, and elevating the risk of mental health deterioration [6].

While the biomedical and economic implications of vision impairment have been widely documented [7,8], its psychological ramifications—particularly its association with depressive symptoms—remain comparatively underexplored. Accumulated evidence suggests that visual decline compromises individuals’ capacity to engage in meaningful daily activities, such as reading, navigating environments, and maintaining interpersonal relationships, thereby increasing the likelihood of emotional distress, social withdrawal, and loss of self-efficacy [9,10]. However, the majority of extant studies tend to emphasize direct associations, often neglecting the internal psychological mechanisms that may mediate, buffer, or modulate the impact of sensory loss on mental health outcomes [11].

In this context, dispositional optimism—defined as the generalized expectation that positive outcomes will occur in the future [12]—emerges as a theoretically and empirically grounded psychological resource. As a stable cognitive-affective trait, optimism has been shown to enhance emotional resilience, promote adaptive coping strategies, and reduce vulnerability to depressive symptoms across diverse life stressors. From a stress-buffering perspective, optimism may attenuate the emotional burden of vision impairment by enabling older adults to reframe sensory decline as manageable rather than debilitating, thereby preserving psychological well-being.

Building on this premise, the present study investigates whether dispositional optimism functions as a mediator in the relationship between vision impairment and depressive symptoms among older Chinese adults. In doing so, we aim to illuminate an understudied yet conceptually significant pathway linking sensory health and emotional outcomes in later life. By situating optimism within a broader framework of cognitive-affective adaptation, the study contributes to ongoing efforts to integrate psychological resilience into public health approaches to aging and sensory loss.

### Theoretical foundations: Dispositional optimism as a psychological mediator

Dispositional optimism—a core component of psychological resilience—fundamentally shapes how individuals perceive, interpret, and respond to adverse experiences [13]. According to the Broaden-and-Build Theory of Positive Emotions, optimism enhances emotional well-being by broadening individuals’ cognitive and behavioral repertoires, thereby promoting adaptive coping strategies in the face of stress [14]. Optimistic individuals are more likely to reframe adversity positively, seek social support, and engage in problem-solving behaviors, mitigating the psychological toll of functional decline. In contrast, those with lower levels of optimism are more prone to maladaptive strategies such as avoidance, rumination, and emotional withdrawal, which can intensify depressive symptomatology [15,16].

The Stress Process Model [17] further conceptualizes optimism as an internal psychological resource that moderates the impact of external stressors, such as vision impairment. Sensory loss threatens autonomy, social integration, and daily functioning; however, optimism can buffer these effects by enabling older adults to reappraise limitations as manageable challenges rather than irreversible losses, thus preserving emotional equilibrium [18].

Empirical research has consistently linked dispositional optimism with reduced depressive symptoms and greater resilience among individuals facing chronic health challenges [19]. Older adults with higher levels of optimism report greater life satisfaction and emotional stability, even in the context of physical impairments [20]. Conversely, reduced optimism has been associated with heightened vulnerability to depressive symptoms, particularly among those experiencing social disengagement or elevated health-related stress [21]. These findings collectively suggest that optimism plays a mediating role by influencing cognitive appraisals, emotional regulation, and behavioral adaptation.

### The vision impairment–depression nexus: a bidirectional dynamic

Importantly, the relationship between vision impairment and depressive symptoms is not unidirectional. Emerging evidence from longitudinal studies indicates a bidirectional dynamic between physical impairment and psychological resilience. Severe or progressive sensory decline may gradually erode an individual’s optimistic outlook, particularly when compounded by disruptions to social routines, independence, or perceived control over one’s environment [22].

Sociocultural factors further mediate this relationship. In collectivist societies such as China, where emotional well-being and personal identity are deeply embedded in family roles and community participation, vision impairment may have outsized psychological impacts. The inability to fulfill intergenerational obligations or participate in communal activities can lead to diminished self-worth and heightened social isolation, thereby undermining optimism and exacerbating depressive symptoms [23]. These consequences are especially pronounced among the oldest-old and individuals in low-resource settings, where access to vision care and psychosocial support remains limited [24]. Such observations highlight the need to attend to both psychological resilience and structural conditions when addressing the mental health impacts of sensory decline.

### Analytical framework and research hypotheses

Drawing on the theoretical foundations discussed above, this study proposes that dispositional optimism mediates the relationship between vision impairment and depressive symptoms among older adults. Anchored in the Stress Process Model [17], the analytical framework underscores that the psychological consequences of sensory decline are contingent not only on the severity of the impairment but also on the presence of internal protective traits.

Dispositional optimism, conceptualized as a stable cognitive-emotional orientation, acts as a stress-buffering mechanism that influences both immediate appraisal and long-term emotional adaptation [13]. Specifically, the study extends the classical Stress-Buffering Hypothesis by identifying two key psychological functions of optimism: (1) Cognitive reframing, whereby optimistic individuals are more likely to perceive impairments as manageable rather than catastrophic, and (2) Emotional regulation, whereby optimism moderates the intensity and duration of negative affective responses.

In contrast to traditional stress-coping models that treat mediators as static buffers, our approach treats optimism as part of a dynamic feedback loop—one in which internal dispositions and external stressors recursively shape each other over time. This conceptualization allows us to test not only whether optimism mediates, but also under what conditions this mediation is more or less effective.

Accordingly, the study puts forward the following hypotheses

**H1 (Stress-Buffer Hypothesis)** Dispositional optimism mediates the relationship between vision impairment and depressive symptoms, such that higher levels of optimism weaken the association by fostering adaptive appraisals and emotional regulation.

**Fig 1:**
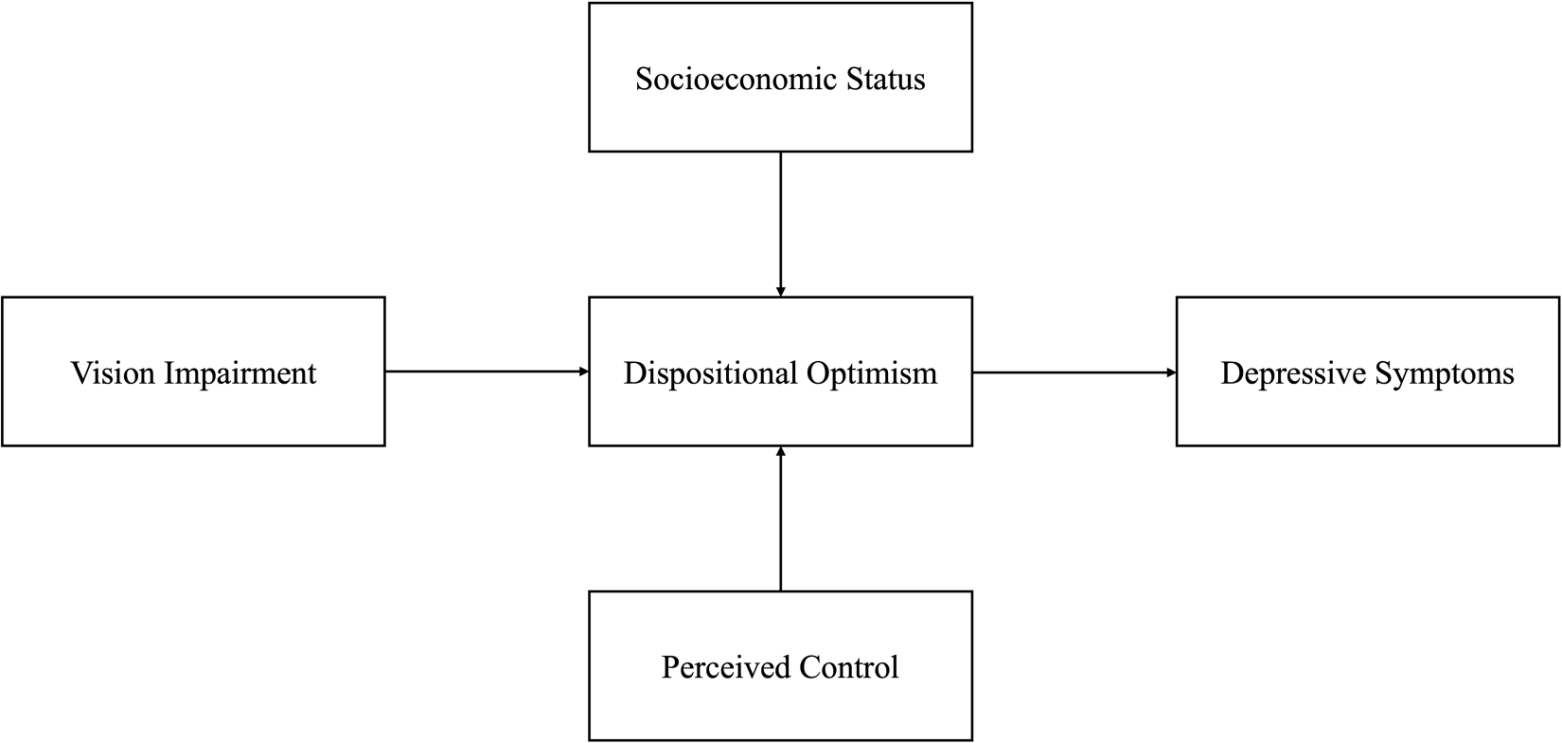
Conceptual framework: dispositional optimism as mediator and contextual buffer. Notes: This framework integrates theories of cognitive-affective adaptation and structural inequality to model emotional resilience in later life. It visualizes the mediating role of optimism and its context-dependent modulation through key social-structural variables.

**H2 (Resource Disparity Hypothesis)** The buffering effect of optimism varies by structural and social conditions, such as socioeconomic status and perceived control over life decisions. Fig 1 shows the study’s conceptual framework.

## Methods

### Data source and sample selection

This study draws on data from the 2014 and 2018 waves of the Chinese Longitudinal Healthy Longevity Survey (CLHLS)—a nationally representative, longitudinal dataset that includes older adults aged 65 and above across 23 provinces in China. The CLHLS adopts a multistage, stratified random sampling design to ensure both demographic and geographic representativeness, capturing respondents from rural, township, and urban areas spanning Eastern, Central, and Western regions. Data were collected via structured face-to-face interviews and objective health assessments, yielding rich information on sociodemographic characteristics, physical and mental health, and psychosocial attributes. The panel structure and breadth of coverage in mental health variables make this dataset particularly suited for investigating depressive symptoms and their determinants among aging populations.

To ensure analytical rigor, we applied strict inclusion and exclusion criteria. Individuals were retained in the final sample if they (1) were aged 65 or older at baseline, (2) participated in both the 2014 and 2018 survey waves, and (3) provided complete data on depressive symptoms, vision impairment, and relevant covariates. Participants with missing or invalid responses on key variables, or with cognitive impairments rendering reliable self-report infeasible, were excluded. After filtering, the final analytical sample comprised 7,205 respondents, providing adequate statistical power for mediation analysis.

### Ethical approval

This study was approved by the Institutional Review Board of Peking University (IRB0001052–13074) and conducted according to the principles outlined in the Declaration of Helsinki and relevant Chinese national laws (Articles 38, 39, and 40 of the Constitution and Article 9, Chapter I of the Statistics Law).

### Informed consent

Verbal informed consent was obtained from all participants prior to interviews. Consent was documented in writing by trained interviewers and witnessed by a second researcher. Participants were informed of their rights, voluntary participation, and confidentiality protections.

### Measures

#### Depressive symptoms.

Depressive symptoms were assessed using the Center for Epidemiologic Studies Depression Scale (CES-D), a widely validated instrument for measuring depressive symptomatology in population-based aging studies [25]. Its cross-cultural applicability has been confirmed in Chinese samples, including within the CLHLS, where studies have demonstrated measurement invariance across subgroups, affirming its cultural validity in a collectivist and gerontological context [26].

Due to instrument variation across waves, harmonization procedures were applied. In the 2014 wave, depressive symptoms were measured using two binary items aligned with the DSM-5 diagnostic criteria for major depressive disorder: (1) prolonged sadness lasting at least two weeks, and (2) marked loss of interest in most activities. Respondents who answered “yes” to both items were coded as having clinically significant depressive symptoms [27,28].

In contrast, the 2018 wave used a 10-item CES-D short form. Respondents rated symptom frequency on a 5-point Likert scale (1 = “never” to 5 = “always”), yielding scores from 10 to 50. A cutoff of ≥30 was used to indicate moderate-to-severe depressive symptoms, consistent with empirical thresholds used in prior studies [29].

To ensure longitudinal consistency, depressive symptoms were dichotomized for both waves: individuals were coded as depressed if their 2014 binary score equaled 2 or their 2018 CES-D score was ≥ 30. This harmonized classification follows established practices in longitudinal mental health research [30].

#### Vision impairment.

Vision impairment was assessed using a field-based functional vision screening adapted from the Landolt C optotype, frequently used in optometric testing [31]. During in-home interviews, participants were shown a circular figure with a directional opening and asked to identify the gap’s direction. The test was conducted under natural indoor lighting, and participants were instructed not to wear corrective lenses—ensuring that the measure captured uncorrected visual function.

Responses were classified into four ordinal categories: (1) Unable to see the circle (severe impairment or blindness); (2) Able to see the circle but unable to identify the direction (moderate impairment); (3) Able to identify the circle but not the orientation (mild impairment); and (4) Able to correctly identify both the circle and its orientation (normal vision).

Although this instrument does not meet clinical ophthalmologic standards (e.g., logMAR or Snellen scales), it offers a functionally meaningful categorization of sensory impairment in everyday settings. For reference, a Snellen visual acuity of 6/18 corresponds approximately to 0.5 logMAR, highlighting the interpretive gap between clinical metrics and field-based functional assessments. While logMAR provides a logarithmic scale of visual resolution and is favored for its statistical precision, our functional screening emphasizes practical limitations in real-world contexts—what we term functional realism—rather than the calibrated granularity of clinical measurement. This distinction is particularly important in large-scale aging surveys where accessibility, feasibility, and ecological validity take precedence over ophthalmologic precision.

The CLHLS vision screening protocol has been widely adopted in Chinese aging research and shown to predict physical, cognitive, and dietary outcomes [32,33], supporting its cultural and contextual validity. Nonetheless, we stress that this measure indicates functional limitations, not clinical diagnoses.

#### Dispositional optimism.

Dispositional optimism—defined as a generalized expectation of favorable life outcomes—was assessed via a single-item measure: “Do you maintain an optimistic outlook on life?” Responses were recorded on a 5-point Likert scale (1 = “strongly disagree” to 5 = “strongly agree”).

While limited in dimensional granularity, single-item optimism measures have demonstrated robust predictive validity and convergent reliability in large-scale studies, especially among older adults, where cognitive load and survey length are important constraints [34,35]. These measures strongly correlate with multi-item tools like the Life Orientation Test–Revised (LOT-R) and are widely used in Chinese aging research due to their feasibility and cultural alignment with collectivist understandings of emotional resilience [36].

#### Covariates.

To control for potential confounding, we included a comprehensive set of demographic, socioeconomic, and contextual variables, each informed by prior research on depression among older adults [37,38].

Age was treated as a continuous variable. Sex was coded dichotomously (0 = female, 1 = male). Marital status was included as a binary variable (0 = unmarried/widowed/divorced; 1 = currently married), capturing relational support. Educational attainment was categorized into: 1 = no formal education; 2 = up to 9 years (primary to middle school); 3 = more than 9 years (high school or above). Economic status was self-rated on a 5-point scale from “very difficult” (1) to “affluent” (5). Household income was grouped into three levels: < 50,000 RMB; 50,000–100,000 RMB; > 100,000 RMB. Residential location was coded as rural (1), township (2), or urban (3). Province of residence was grouped into Western (1), Central (2), or Eastern China (3). Perceived control over life decisions, a key psychosocial variable, was measured on a 5-point Likert scale (1 = “no control at all” to 5 = “full control”), reflecting individual agency and autonomy. These covariates were included in all regression models to isolate the net effects of vision impairment and optimism on depressive symptoms.

### Statistical analysis

All statistical analyses were conducted using Stata 16.0 (StataCorp, College Station, TX). Descriptive statistics were generated to summarize the distribution of key variables. Variance inflation factor (VIF) tests confirmed the absence of multicollinearity among covariates.

To estimate the association between vision impairment and depressive symptoms, we employed random-effects (RE) ordered logit regression, accounting for both within- and between-individual variation. Subgroup analyses were conducted to assess heterogeneity by perceived control, dividing the sample into high and low control groups.

The mediation effect of dispositional optimism was tested using bootstrapped structural equation modeling (SEM) with 1,000 replications to obtain robust confidence intervals for indirect effects [41,42].

Robustness checks using Probit models and alternative outcome specifications produced consistent results. Additionally, the mediation pathways remained stable across perceived control subgroups, enhancing the generalizability and internal consistency of the findings.

## Results

### Descriptive analysis

The descriptive statistics (Table 1) illustrate the distinctive vulnerabilities of the study population. The sample had a mean age of 90.53 years (SD = 9.88), ranging from 65 to 123, highlighting its advanced aging profile. This demographic distribution underscores the cohort’s heightened susceptibility to sensory decline, as evidenced by the mean vision impairment score of 3.56 (SD = 0.76) on an 8-point functional scale, indicating moderate impairment. Compared to younger adults, individuals in this age group are less likely to undergo corrective procedures due to surgical risks, constrained access to care, or age-related cultural norms surrounding medical intervention [39]. Many older adults instead adapt through silent endurance, making it vital to understand their coping resources within a broader psychosocial context.

**Table 1:** Descriptive statistics.

Variable	Mean	Standard Deviation	Min.	Max.	Observations
Depressive symptoms	0.077	0.266	0	1	7,205
Vision impairment	3.556	0.759	1	8	7,205
Dispositional optimism	3.902	0.654	1	5	7,205
Perceived control over life decisions	3.084	1.243	1	5	7,205
Sex	0.506	0.5	0	1	7,205
Age	90.532	9.879	65	123	7,205
Province of residence	2.242	0.805	1	3	7,205
Marital status	0.464	0.499	0	1	7,205
Education	1.861	0.792	1	3	7,205
Economic status	3.104	0.627	1	5	7,205
Household income	1.36	0.532	1	3	7,205
Residential location	1.603	0.721	1	3	7,205

Most respondents reported a relatively positive outlook on life (mean optimism = 3.90, SD = 0.65), yet the mean perceived control score of 3.08 (SD = 1.24) reveals substantial heterogeneity in perceived autonomy. This variability suggests that optimism may function as an internal resource for emotional adaptation in contexts where control is constrained.

Socioeconomic indicators further contextualize psychological vulnerability. The average education level (1.86) reflects limited formal schooling, while the household income level (1.36) indicates modest financial resources. These structural constraints may exacerbate exposure to depressive symptoms, reinforcing the need to analyze both personal resilience and environmental stressors.

### The impact of vision impairment on depressive symptoms

A variance inflation factor (VIF) test confirmed the absence of multicollinearity (mean VIF = 1.16), ensuring model reliability. Results from the ordered logistic regression models (Table 2) demonstrate a robust relationship between vision impairment, optimism, and depressive symptoms.

**Table 2:** Ologit regression results.

Variable	Step 1 (DV: Depressive symptoms)	Step 2 (DV: Depressive symptoms)	Step 3 (DV: Dispositional optimism)
Vision impairment	−0.218*** (−3.63)	−0.173** (−2.86)	0.052*** (5.02)
Dispositional optimism		−0.914*** (−13.05)	
Sex	−0.492*** (−4.73)	−0.421*** (−4.02)	0.057*** (3.45)
Age	−0.007 (−1.34)	−0.004 (−0.75)	0.003** (2.82)
Marital status	−0.317** (−2.86)	−0.285** (−2.56)	0.049** (2.78)
Education	0.240*** (3.83)	0.264*** (4.12)	0.026** (2.59)
Economic status	−0.958*** (−12.23)	−0.723*** (−9.16)	0.210*** (16.99)
Household income	−0.022 (−0.24)	−0.022 (−0.23)	0.011 (0.72)
Province of residence	−0.184*** (−3.18)	0.3082	0.075*** (7.84)
Residential location	0.090 (1.34)	0.120 (1.78)	0.048*** (4.50)

Notes: (1) Step 1: Depressive symptoms predicted by vision impairment and other covariates. (2) Step 2: Depressive symptoms predicted by vision impairment, dispositional optimism, and covariates. (3) Step 3: Dispositional optimism predicted by vision impairment and covariates. (4) ***p < 0.001, **p < 0.01, *p < 0.05. (5) Coefficients are displayed with z-statistics in parentheses.

In Step 1, vision impairment significantly predicted higher depressive symptoms (β = −0.218, p < 0.001), supporting the notion that better visual function is associated with improved mental health.

In Step 2, dispositional optimism was negatively associated with depressive symptoms (β = −0.914, p < 0.001). After accounting for optimism, the coefficient for vision impairment was attenuated (β = −0.173, p < 0.01), suggesting partial mediation.

In Step 3, vision impairment was positively associated with optimism (β = 0.052, p < 0.001), which may indicate a resilient subgroup of older adults who maintain psychological resources despite physical decline.

### Moderating role of perceived control

To test for heterogeneity, the sample was stratified by perceived control using a cutoff score of 3, based on Self-Determination Theory [40]. Results (Table 3) show that:

**Table 3:** Heterogeneity test results.

Variable	Lower Perceived Control (2,373)	Higher Perceived Control (4,832)
Vision impairment	−0.193 (−1.20)	−0.135* (−2.10)
Dispositional optimism	−0.802*** (−4.87)	−0.870*** (−12.42)
Sex	−1.242*** (−4.54)	−0.247* (−2.22)
Age	0.025 (1.92)	−0.011 (−1.74)
Marital status	0.542 (1.90)	−0.407*** (−3.39)
Education	0.753*** (4.97)	0.129 (1.90)
Economic status	−0.398 (−1.81)	−0.693*** (−8.49)
Household income	−0.095 (−0.42)	0.030 (0.28)
Province of residence	−0.025 (−0.18)	−0.159* (−2.50)
Residential location	−0.010 (−0.06)	0.150* (2.02)

Notes: (1) Lower Perceived Control: Results from the subgroup with autonomy scores < 3. (2) Higher Perceived Control: Results from the subgroup with autonomy scores ≥ 3. (3) ***p < 0.001, **p < 0.01, *p < 0.05. (4) Coefficients are presented with z-statistics in parentheses.

First, among respondents with **low perceived control**, vision impairment was not significantly associated with depressive symptoms (β = −0.193, *p* = 0.230), suggesting a potential mechanism of **learned helplessness**. Yet, optimism still exerted a strong protective effect (β = −0.802, *p* < 0.001).

Second, among those with high perceived control, both vision impairment (β = −0.135, p < 0.05) and optimism (β = −0.870, p < 0.001) significantly predicted depressive symptoms, supporting the context-dependent buffering effect of optimism.

### Mediation analysis: the role of dispositional optimism

Mediation results (Table 4) support the Stress-Buffering Hypothesis. Optimism accounted for 24.7% of the total effect of vision impairment on depressive symptoms. Bootstrapping with 1,000 replications confirmed the statistical significance and robustness of the indirect effect.

**Table 4:** Bootstrapping mediating statistics.

Effect	Coefficient	Std. Err.	LLCI	ULCI	Ratio
Total effect (c)	−0.016	0.004	−0.025	−0.008	
Direct effect (c’)	−0.012	0.004	−0.021	−0.003	
Indirect effect (ab)	−0.004	0.001	−0.006	−0.002	
Proportion of total effect mediated					**0.247**
Ratio of indirect to direct effect					**0.328**
Ratio of total to direct effect					**1.328**

Notes: (1) Abbreviations: LLCI, lower level for confidence interval; ULCI, upper level for confidence interval. (2) Total effect (c): The overall association between vision impairment (IV) and depressive symptoms (DV). (3) Direct effect (c’): The effect of vision impairment on depressive symptoms when controlling for the mediator (dispositional optimism). (4) Indirect effect (ab): The portion of the effect mediated by dispositional optimism. (5) Proportion of total effect mediated: Indicates the percentage of the total effect explained by the indirect pathway. (6) Ratios: Show the relative contribution of the indirect effect compared to the total and direct effects.

These findings suggest that optimism mitigates the psychological salience of sensory decline rather than reversing its occurrence—highlighting the importance of internal traits in mediating environmental stressors.

### Robustness test

Robustness checks (Table 5) confirm the consistency of findings. The random-effects Probit model yielded comparable results. Mediation remained significant in both high and low perceived control groups, although stronger in the latter, where the direct pathway was non-significant.

**Table 5:** Robustness test statistics.

Model	Independent Variable	Coefficient	Std. Error	P-value	95% Confidence Interval	N	Method
Random-Effects Probit Regression	Vision impairment	−0.113**	0.031	0.000	[-0.174, -0.052]	7,205	Probit regression
Mediation Analysis (High Control Group)	Indirect effect	−0.0036**	0.0011	0.0017	[-0.0058, -0.0014]	4,832	Sobel-Goodman mediation test
Mediation Analysis (High Control Group)	Direct effect	−0.011*	0.0055	0.042	[-0.022, -0.0004]	4,832	Sobel-Goodman mediation test
Mediation Analysis (Low Control Group)	Indirect effect	−0.0017*	0.0007	0.012	[-0.0031, -0.0004]	2,373	Sobel-Goodman mediation test
Mediation Analysis (Low Control Group)	Direct effect	−0.006	0.0055	0.243	[-0.017, 0.004]	2,373	Sobel-Goodman mediation test

Together, these results affirm that dispositional optimism consistently mediates the link between sensory decline and mental health, even in the presence of low perceived control—highlighting its relevance as a resilience-enabling resource in the aging process.

## Discussion

This study offers compelling evidence that vision impairment functions not only as a biomedical constraint but as a psychosocial stressor, with meaningful implications for mental health in late life. By empirically testing the mediating role of dispositional optimism, the study elucidates a psychological mechanism through which sensory decline translates into depressive symptomatology—while also revealing how this mechanism varies across levels of perceived control and socioeconomic advantage.

These findings carry theoretical, empirical, and policy relevance, particularly in aging societies undergoing rapid structural transformation and rising disparities in health and psychological resources.

### Vision impairment and depression: a multi-layered stressor

Our findings reinforce the claim that vision impairment is not a unidimensional sensory loss but a multifaceted life disruption. Statistically, older adults with worse vision had a significantly higher probability of experiencing depressive symptoms (β = −0.218, p < 0.001), consistent with the Stress Process Model [17], which conceptualizes stress as the product of an individual’s exposure to environmental demands and their available coping resources.

This vision-related stress unfolds across three levels:

First, instrumental level. Impaired vision reduces the capacity to perform basic and instrumental activities of daily living (e.g., reading medication labels, walking safely, recognizing facial cues), which leads to a cumulative erosion of functional autonomy.

Second, social level. Sensory loss undermines relational embeddedness, making it difficult for older adults to sustain conversations, participate in community rituals, or maintain intergenerational bonds. This is especially salient in collectivist cultures like China, where older adults’ social value is often anchored in their relational utility and ceremonial participation [43].

Third, existential level. Vision impairment disturbs narratives of aging with dignity. When sensory decline is experienced as irreversible, and when its effects are inadequately acknowledged by younger family members or health systems, it can generate ontological insecurity—a silent but persistent sense that one is being gradually erased from social visibility and moral relevance [1–7].

These three levels work in concert to erode self-efficacy, emotional stability, and ultimately, mental well-being, particularly for the oldest-old and those living in structurally disadvantaged contexts.

### Dispositional optimism: mediator and meaning-maker

The study reveals that dispositional optimism accounts for 24.7% of the total effect linking vision impairment and depression (indirect effect = −0.004, p < 0.01). This suggests that optimism—far from being a passive personality trait—is a mediating force that actively shapes how older adults internalize and respond to sensory decline.

From a theoretical perspective, dispositional optimism acts on three mutually reinforcing dimensions:

First, cognitive dimension. It enables reappraisal of loss events. Optimistic individuals are more likely to perceive impairment not as an endpoint, but as an adaptive challenge—potentially manageable through rehabilitation, accommodation, or spiritual reframing [12,13].

Second, affective dimension. Optimism helps sustain positive emotional tone (hope, gratitude, anticipation), consistent with the Broaden-and-Build Theory [14]. These positive states are not merely buffers against despair but serve as resources for meaning-making, helping older adults anchor their experiences within long-term life narratives.

Third, behavioral dimension. Optimism is linked to activation energy. It motivates action—e.g., seeking assistive devices, reaching out to friends, or continuing to participate in family events even with limitations [15,16]. In contrast, pessimism tends to correlate with emotional inertia and behavioral withdrawal.

This finding affirms the constructive function of optimism in shaping aging trajectories: it mediates not just between vision loss and depression, but between loss and the preservation of meaning.

### The moderating role of perceived control: The conditionality of optimism

A critical insight from the subgroup analysis lies in the context-dependency of optimism’s buffering effect. Among those with high perceived control, vision impairment retained a direct effect on depression (β = −0.135, p < 0.05), but optimism significantly mediated this effect (indirect effect = −0.0036, p < 0.01). Conversely, among those with low perceived control, vision impairment no longer had a significant direct effect, but optimism still buffered depression (indirect effect = −0.0017, p < 0.05).

This asymmetry suggests two things:

On the one hand, for high-control individuals, vision loss may threaten their sense of mastery, making them more sensitive to functional decline. Optimism in this context preserves internal coherence, allowing them to reinterpret loss without compromising self-efficacy.

On the other hand, for low-control individuals, the psychological threat posed by vision loss may be less salient, as a generalized sense of helplessness is already pervasive. Yet, optimism still exerts a counterforce, preventing complete resignation.

This finding supports a dynamic model of resilience [11,12], wherein optimism functions not as a universal shield but as a context-responsive resource—its effectiveness shaped by the degree to which individuals feel they can shape their own futures.

### Sociocultural embeddedness: The Chinese context

The role of optimism is deeply entangled with sociocultural logics. In China’s familistic and duty-bound aging culture, optimism is often expressed not as personal aspiration but as relational hope—the belief that one’s family will be well, that obligations will be fulfilled, and that harmony will be maintained even amid decline [43–45].

Vision loss in this context can disrupt symbolic roles, such as ancestor worship, caring for grandchildren, or managing household rituals. These are not trivial activities but central to one’s moral selfhood. Optimism, then, does not merely buffer emotional pain; it restores continuity between the impaired present and the meaningful past.

Structural variables such as education (β = 0.264, p < 0.001) and economic status (β = 0.210, p < 0.001) significantly predicted optimism, showing how material conditions scaffold emotional orientation. In this sense, optimism is not just a personal disposition but a socially distributed asset, unevenly shaped by access to resources and supportive institutions.

### Theoretical and practical implications

This study extends the Stress Process Model [17] by conceptualizing optimism as an active psychological mediator, not merely a passive trait or static moderator. It foregrounds the processual nature of stress-buffering: how internal traits and external conditions continuously interact to shape emotional trajectories. By doing so, the study moves beyond linear cause-effect models toward a relational, temporally dynamic understanding of resilience.

Practically, these findings suggest that vision rehabilitation programs must go beyond biomedical correction. They should incorporate psychological screening and strength-based interventions that cultivate dispositional optimism. For instance: (1) Cognitive-behavioral training adapted for older adults with sensory impairments; (2) Group-based narrative therapy that affirms individual dignity and social worth; (3) Community programs that reinforce perceived control through participatory decision-making.

Particular attention should be paid to resource-scarce environments, where structural disadvantages intersect with sensory decline. In such settings, cultivating optimism may serve as a low-cost, scalable intervention to prevent mental health deterioration.

### Limitations and directions for future research

Crucially, the visual acuity measurement used in this study—though widely adopted in aging surveys [31–33]—diverges from gold-standard ophthalmologic procedures. It should be interpreted not as a precise clinical diagnostic but as a pragmatic proxy for everyday visual functioning under lived conditions. This limitation reflects a broader epistemological tension in survey-based gerontology: between clinical realism, which demands calibrated precision, and functional realism, which privileges embodied, socially situated impairments. Future studies may benefit from combining clinical visual assessments with qualitative or ethnographic approaches to better capture older adults’ visual agency and coping strategies.

Similarly, the use of a single-item measure to assess dispositional optimism, while supported by large-scale studies in geriatric contexts [34–36], lacks dimensional granularity. It may not fully capture the dual cognitive (e.g., generalized expectancies) and affective (e.g., emotional tone) components of optimism, thereby introducing potential measurement error and construct attenuation. The mediating role of optimism reported here should thus be considered a conservative estimate. Future research would benefit from applying multi-item instruments such as the Life Orientation Test–Revised (LOT-R), and from examining how optimism interacts with related constructs like hope, perceived control, and future self-continuity.

Beyond these measurement considerations, several other limitations merit acknowledgment. First, depressive symptoms among older adults—particularly in collectivist and high-stigma contexts like China—may be underreported due to cultural norms of emotional restraint and generational taboos surrounding mental illness. This potential social desirability bias may dampen the observed associations. Second, longitudinal attrition between survey waves—especially among participants with severe health conditions or institutionalization—could bias the results toward healthier or more resilient survivors, thereby limiting representativeness. Third, the generalizability of our findings beyond the Chinese context remains uncertain, as cultural scripts around aging, optimism, and intergenerational roles may differ substantially across societies. Replication in other cultural settings is essential to validate the robustness and universality of the observed mechanisms.

Fourth, we were unable to account for potentially important unmeasured moderators such as chronic disease burden, social support networks, or household caregiving structures. These variables may shape both the psychological impact of vision impairment and the protective effects of optimism. Finally, while our analysis focuses on optimism as a key mediating mechanism, we recognize that other psychological processes—such as perceived stress, coping styles, or meaning-making—may also play critical roles in linking sensory decline to depressive outcomes. Expanding the mediation model to incorporate these alternative pathways would provide a more comprehensive understanding of resilience in later life.

## Conclusion

This study advances a more nuanced understanding of how sensory health intersects with emotional resilience in later life by demonstrating that dispositional optimism mediates the psychological consequences of vision impairment. Rather than treating optimism as a stable personality trait or a background control variable, we situate it as a structurally conditioned and socially embedded mediator—one that responds to both lived impairment and contextual inequalities.

Through a multi-level analytic framework, this research contributes to the growing field of psychosocial gerontology by foregrounding three conceptual insights:

First, it reconceptualizes vision impairment not as a biomedical endpoint, but as a catalyst for adaptive or maladaptive psychological responses, contingent on available cognitive-affective resources.

Second, it operationalizes optimism as a dynamic mediational process, empirically measurable yet theoretically sensitive to variations in perceived control and socioeconomic conditions.

Third, it offers a portable model—one that can be tested across diverse aging populations and adapted to culturally specific forms of resilience and vulnerability.

Methodologically, the study integrates harmonized multi-wave survey data with robust mediation models, offering a replicable approach to examining psychological buffering mechanisms within structural constraint. Its findings underscore the importance of combining quantitative rigor with cultural reflexivity in aging research.

Looking forward, future research would benefit from tracing temporal shifts in optimism, examining how it is reinforced or eroded through repeated exposure to sensory loss, relational dislocation, or institutional neglect. Additionally, expanding the framework to include other affective resources—such as compassion, trust, or future self-continuity—would help clarify how older adults mobilize psychological meaning under conditions of structural stress.
